# HEARTS in the Americas: a global example of using clinically validated automated blood pressure devices in cardiovascular disease prevention and management in primary health care settings

**DOI:** 10.1038/s41371-022-00659-z

**Published:** 2022-02-24

**Authors:** Pedro Ordunez, Cintia Lombardi, Dean S. Picone, Tammy M. Brady, Norm R. C. Campbell, Andrew E. Moran, Raj Padwal, Andres Rosende, Paul K. Whelton, James E. Sharman

**Affiliations:** 1 Department of Non-Communicable Diseases and Mental Health, Pan American Health Organization, Washington, DC USA; 2 Menzies Institute for Medical Research, University of Tasmania, Hobart, TAS Australia; 3 Department of Pediatrics, Division of Nephrology, Johns Hopkins University School of Medicine, Baltimore, MD USA; 4 Departments of Medicine, Physiology and Pharmacology and Community Health Sciences, Libin Cardiovascular Institute of Alberta, Calgary, AB Canada; 5 Resolve to Save Lives, New York City, NY USA; 6 Department of Medicine, University of Alberta, Edmonton, AB Canada; 7 Department of Epidemiology, Tulane University School of Public Health and Tropical Medicine, New Orleans, LA USA

**Keywords:** Hypertension, Risk factors

## Introduction

Cardiovascular disease (CVD) is the leading cause of illness burden in the Americas, causing over 2 million deaths in 2017, a third of deaths overall for that year. While striking, CVD is threatening to have an even greater impact in the coming years based on the upward trend of the last decade [1]. Raised blood pressure (BP), a significant risk factor for the development of CVD, causes over 50% of ischemic heart disease and stroke events and 17% of total deaths globally [2]. The prevalence of hypertension (defined as SBP/DBP ≥140/90 mmHg or taking antihypertensive drugs) in Latin America and the Caribbean is 28% in women and 43% in men [3]. Thus, effective identification and treatment of hypertension is central to CVD prevention and decreasing CV morbidity and mortality [4].

To address the growing burden of CVD, the Pan American Health Organization (PAHO) initiated HEARTS in the Americas, a comprehensive risk reduction initiative set to become the institutionalized model for hypertension management in primary health care by 2025. It is a multinational initiative, led by the Ministries of Health from each participating country, and with the participation of local stakeholders and the technical cooperation of PAHO. Key outcomes include improvements in detection, treatment, and control of hypertension in each population served [5]. Currently, the initiative is being implemented and expanded in 915 primary health care facilities located in 20 countries (https://www.paho.org/en/hearts-americas).

Accurate BP measurement is key to effective diagnosis and management of hypertension and is one of the six strategic pillars of HEARTS in the Americas initiative. (https://www.paho.org/en/hearts-americas).

Accurate BP measurement requires proper patient preparation, an appropriate setting, a standardized measurement protocol and use of a clinically validated automated BP measuring device (BPMD). The problem of uncontrolled hypertension at the global level is huge and multifactorial and the issue of non-validated BPMD is an important factor. According to the Lancet Commission on Hypertension Group, the accuracy of BP measurement is a serious and frequent problem of contemporary clinical practice that should be considered as an issue of patient safety. Indeed, the Commission has alerted from about 3000 cuff-based BPMDs on the market today, less than 15% have published evidence on accuracy performance. These non-validated BP devices used in clinical practice are more likely to be inaccurate [6]. Moreover, in most of the countries implementing HEARTS in the Americas, the current regulatory landscape is weak and fragmented that means there is a limited scope to prevent the use of non-validated BPMDs. The lack of policies and regulations promoting the exclusive use of clinically validated automated BPMDs is a significant impediment to the success of the initiative [7].

This perspective article outlines collaborative work between PAHO, implementing countries and partners to improve the regulatory landscape related to BPMDs in countries implementing HEARTS. First, the WHO/PAHO position on ensuring the quality of medical devices, including BPMDs will be described. Second, the relevance of regulating market approval and procurement of clinically validated automated BPMDs and associated policy implications will be highlighted. Third, the strategies to implement the exclusive use of clinically validated automated BPMDs will be detailed. Guiding principles are that improving the regulatory framework and procurement mechanisms will contribute to the exclusive use of clinically validated automated BPMDs in primary health care facilities by 2025. This achievement will enhance the HEARTS in the Americas initiative to decrease the burden of CVD in the Americas.

## The World Health Organization and PAHO position on medical devices and BPMDs

The Fourth WHO Global Forum on Medical Devices recommended specific actions to increase the regulation of medical devices and to optimize the procurement process in low- and middle-income countries [8]. The WHO declared BPMDs as one of seven general medical device priority areas requiring assessment [9]. Moreover, in 2020, the WHO published an updated guidance document regarding BPMDs in response to concerns about the lack of accurate, high-quality devices, especially in low- and middle-income countries [10].

Likewise, in 2015, the PAHO Directing Council recommended that governments prioritize the development of legal and organizational frameworks to oversee the quality, safety, and efficacy of health technologies available on the market [11]. Accordingly, PAHO determined that many countries have limited or no legal and organizational framework for the regulation of medical device sales, an issue highly relevant to BPMDs [12]. These WHO and PAHO documents are key policy instruments that frame the HEARTS in the Americas regulatory pathway and enable exclusive use of clinically validated automated BPMDs across participating countries. However, more work is needed to ensure the successful implementation of these policies.

## Regulating market approval and procurement of validated BPMDs

Regulation is critical to ensure that safe, rigorously tested medical devices are used in health care. Regulations of BPMDs should address accuracy and precision as a crucial component of quality assurance. Accuracy and precision can be verified through the successful completion of an independently conducted clinical validation study using an accepted validation protocol [13]. Certainly, if health facilities, health providers or patients purchase non-validated BP devices, those not proven accurate according to internationally accepted standards, hypertension management may be based on inaccurate readings. However, most automated BPMDs currently available for purchase have not been clinically validated for accuracy and precision. For instance, one study from Australia showed that <20% of BPMDs were clinically validated [14]. Barriers to conducting validation studies include cost [15] and required technical expertise. Until governments mandate validation testing according to international scientific standards prior to market approval, manufacturers will continue to have little incentive to overcome these barriers.

One factor that may facilitate a move toward exclusive use of clinically validated automated BPMD is advocacy by professional societies and academic leaders working to improve hypertension control. For example, in 2016, World Hypertension League and partners called to regulate the manufacture and marketing of BPMD [16]. In 2020, the Lancet Commission on Hypertension Group recommended a mandatory global regulatory requirement that BPMDs successfully complete independent clinical validation according to the universally accepted ISO Standard (ISO 81060-2:2018) prior to marketing and use [6]. HEARTS in the Americas is working to promote those recommendations, and to our knowledge is the first multinational program in the world to do so.

It is important to note that regulating market approval and requiring the procurement of clinically validated automated BPMDs globally has clear policy implications. For instance, the implementation of a mandatory regulatory policy may increase the cost associated with the development and manufacture of BPMDs, resulting in higher health care costs. On the other hand, these regulations would reduce unfair competition from companies who do not have their devices validated, which may enhance the production of higher quality devices overall. Despite the theoretical concerns, by increasing the availability of validated BPMDs for clinical use, hypertension control may be optimized, decreasing health care costs over time. A further evaluation to better understand the global cost-benefits of a mandatory validation before market approval might be needed.

## HEARTS in the Americas: strategies toward the exclusive use of automated validated BPMDs

The strategies promoted by HEARTS in the Americas to implement the exclusive use of validated BPMDs in primary health care are manifold.

### Understanding the regulatory context

A pivotal action to facilitate the progress of the HEARTS in the Americas agenda was to examine the regulatory frameworks related to BPMDs in participating countries. A recent study [7] found that most countries (10 of 13) had an existing medical devices law, or the foundation for such a law, that stipulated a regulatory bodies’ responsibilities. However, only six countries had regulations that applied explicitly to BPMDs, and just two had regulations that mandated accuracy and precision validation criteria as a condition of pre-market approval. Seven countries had government bodies responsible for enforcing medical device regulations, and seven had mechanisms to remove devices from the market that did not comply with regulations. Seven countries had a centralized system of acquiring BPMDs, but the acquisition was only regulated in six of these countries. In summary, BPMDs can be approved for sale in most countries without the manufacturer providing evidence of passing an internationally accepted clinical validation standard.

### Creating awareness

This topic was introduced by a thematic HEARTS webinar and was addressed in the first HEARTS regional technical meeting in the Dominican Republic in 2019. In March 2020, a technical meeting with participating regulatory authorities and Ministries of Health was conducted in Ecuador to review survey results regarding regulations and explore actions to improve the situation. Several bilateral meetings with HEARTS implementing countries were conducted. Finally, a webpage explicitly devoted to materials related to BP measurement resources was developed (https://www.paho.org/en/hearts-americas/hearts-americas-blood-pressure-measurement). The term “validated” became part of the HEARTS lexicon associated with BPMDs and is used in all promotional materials.

### Training on protocols for BPMD validation

Training on how to conduct clinical validation studies is necessary to build national and regional capacity to test devices and facilitate a broader understanding of clinical validation. This is particularly relevant to countries that manufacture BPMDs for their local market, rather than just importing BPMDs (e.g., Brazil and Cuba). A workshop was conducted in Cuba to train academics and health professionals to perform validation of a locally produced BPMD. In the Americas, only Brazil, Canada, Cuba, and the US, produce BPMDs for clinical use.

### Providing information on validated BPMDs

Despite the growing commitment of countries participating in HEARTS in the Americas to use clinically validated BPMDs, finding the resources to identify and purchase validated BPMDs has been challenging. Thus, a list of organizations with websites that list validated BPMDs was created (https://www.paho.org/en/documents/lists-validated-automated-blood-pressure-measuring-devices). While not specifically endorsed by PAHO, countries also have access to validated device listing websites hosted by different organizations across the globe to help determine if a device of interest has passed clinical validation testing [17]. A list focused on automated validated BPMDs available for sale in the region of the Americas for clinical use is currently under development by PAHO.

### Development of technical specifications for procurement of BPMDs

The PAHO Technical Specifications (https://www.paho.org/en/hearts-americas/hearts-americas-blood-pressure-measurement) for device procurement have been adapted from the WHO report on technical specifications [10] and is being used for procurement of BPMDs by the PAHO Strategic Fund. The strategic fund is a regional technical cooperation mechanism for the pooled procurement of essential medicines and strategic health supplies (https://www.paho.org/en/paho-strategic-fund).

### Advocacy for the implementation and modification of regulations for BPMDs

Regulations can be promoted as a tool for ensuring patient safety, improved health care quality, promotion of a team-based care approach, and a way to meet a global standard of care. Support by key stakeholders, such as professional societies and academia, can significantly facilitate the implementation of regulations if viewed as an improvement in health care practice [17]. For instance, HEARTS in the Americas has developed a framework to support implementing countries on the regulatory pathway to the exclusive use of clinically validated automated BPMDs (https://www.paho.org/en/hearts-americas/hearts-americas-blood-pressure-measurement).

### Participatory process and gradual implementation

Developing and implementing regulations toward the exclusive use of automated validated BPMDs is an urgent need but this must be a well-planned, progressive, inclusive, and participatory process. Implementation must be gradual and phased in to facilitate acceptance, provide time for a realistic substitution of BPMDs, avoid high costs, and prevent challenges for manufacturers and distributors. To facilitate this process and collaborate with implementing countries, PAHO has developed a document that focuses on measures to improve regulatory frameworks (https://www.paho.org/en/hearts-americas/hearts-americas-blood-pressure-measurement). Table 1 shows a summary of these recommendations.

**Table 1:** HEARTS in the Americas: recommendations toward the exclusive use of clinically validated, automated BPMDs.

Recommendations	Description
Early adoption strategy	The government acts to regulate the market instead of waiting for voluntary measures by the manufacturers and distributors (self-regulation). This strategy may motivate the industry to promote BPMDs that have been validated and thus use them as a marketing strategy, generating a win-win situation.
Gradual implementation	To facilitate acceptance and provide time for a realistic substitution of automatic validated BPMDs, including avoiding high costs, preventing challenges by manufacturers and distributors, regulations must be designed to allow for implementation over time. A grace period for procurement may help in that process.
Timeframe	Establishing a timeframe to debate and adopt the regulation, and fully implement it may reduce the potential for the regulation lagging. Defining a specific timeframe helps identify the goals and comply with the commitments of the parties involved.
Flexibility	It may be hard to change medical devices laws, but the regulation must be flexible to allow for modifications and adjustments as needed. New standards for accuracy validation and other scientific evidence, when available, must be incorporated swiftly to increase its effectiveness. A system to recognize standards faster than modifying regulations should be adopted to include a rapid revision of international standards.
Sustainability	Regulation must be a component of a national health plan and strategy for NCD, cardiovascular health/hypertension, and medical devices laws.
Monitoring and evaluation	To measure the impact of the implementation of a regulatory framework and correct its course, the implementation should be monitored and evaluated. This process must be linked to the procurement of BPMDs for use in primary health care facilities. HEARTS system for monitoring and evaluation already includes BPMDs and certification to BP measurement among its indicators.
Enforcement	Mechanisms, roles of responsible government bodies, and budget for monitoring and enforcement of compliance, including sanctions, need to be defined. In addition, coordination with other government sectors (e.g., Trade, Customs) must be set in place, mainly since most BPMDs are imported into the Americas.

### Incorporation into more comprehensive BP measurement capacity building and other activities

HEARTS has also worked to improve other aspects important to accurate BP measurement, including clinical training. The use of clinically validated devices remains key to accurate measurement. As a result, many primary health care facilities implementing HEARTS have prioritized both the training and certification of their personnel in the correct measurement of BP [18] and the availability of clinically validated automated BPMDs.

## Conclusion

The HEARTS in the Americas approach to ensuring accuracy BP measurement, which includes increasing awareness, advocacy, training, and certification of primary health care personnel and the adoption of a regulatory framework that mandates the exclusive use of clinically validated automated BPMDs, is being progressively deployed. This initiative has become a regional game-changer and a significant incentive to accelerate a smooth transition toward the exclusive use of clinically validated automated BPMDs in the primary health care settings by 2025 in the Latin America and Caribbean region. Thus, HEARTS in the Americas is a global exemplar of a strategic journey to improve hypertension control and CVD management.
